# Houdini’s Illusions: Some Acts Are Not What They Seem to Be

**DOI:** 10.2967/jnumed.121.262631

**Published:** 2021-12

**Authors:** Roelof J. Bennink, Mathias Prokop, Andor W. J. M. Glaudemans, Liesbeth Peters-Bax, Jasper H. G. Helthuis, Arthur Adams, Peter R. Kornaat, Adriaan A. Lammertsma, Lenka M. Pereira Arias-Bouda

**TO THE EDITOR:** In The Netherlands, the residency training programs for nuclear medicine and radiology were merged in 2015. This integrated residency training program was born from the vision that clinicians should not have to deal with multiple imaging specialists who are modality-based and partially knowledgeable but rather with a single imaging specialist who is knowledgeable with respect to all imaging modalities utilized to answer a clinical question. This change not only facilitated and improved the interpretation of hybrid imaging but also paved the way toward deeper understanding of disease processes, better therapies, and increased cost-effectiveness. The nuclear radiologist (nuclear medicine and molecular radiology) is our next-level nuclear medicine physician.

The paper of Velleman et al. (*1*) provides a snapshot, taken mid-2020, based on a sample of less than one third of Dutch residents, with only 9 respondents from the nuclear medicine subspecialty program. Because of these small numbers, the results have to be interpreted with care. Velleman et al. correctly state that only 14 new residents had chosen the nuclear medicine differentiation in April 2020, down from over 50 in 2015. This information prompted an editorial (*2*) pointing out that “Because of this more than 70% decrease within just 5 y, the question is not ‘if’ but ‘when’ nuclear medicine as a strong and innovative discipline will disappear in The Netherlands.” These numbers, together with the alarming editorial, underscore the need for further analysis and critical appraisal of the situation.

Within the new 5-y Dutch curriculum, residents choose their subspecialty (e.g., nuclear radiology) after completion of 2.5 y of general training in imaging. At that time, their choice is registered. This fact alone reduces the number of registered residents in nuclear medicine by a factor of 2, as half the residents involved in the new curriculum have simply not recorded their choice yet.

From the start of the new training program, the number of residents in this curriculum was small: 10 were registered in April 2019, growing to 14 (the number mentioned in the article) in April 2020 and 26 in April 2021. These residents have gradually replaced residents from the former dedicated nuclear medicine program.

Moreover, the total number of resident positions in The Netherlands (all specialties), determined yearly by a commission of the Dutch government for each medical specialty, has shrunk substantially over the past few years. Since the all-time high in 2015, the total number of residents in medical imaging has decreased by 20%. As a result, the percentage of all residents choosing nuclear medicine now, in fact, exceeds the percentage in 2015.

We do agree with Czernin and Herrmann (*2*) that The Netherlands has a proud history in the development of nuclear medicine, to which Dutch scientists made a major contribution. We also agree that nuclear medicine is much more than hybrid imaging, and we strongly believe in the multidisciplinary nature of our field. Interestingly, even the small numbers mentioned in the paper of Velleman et al. (*1*) show a tendency for residents who chose nuclear medicine to be more academic and interested in research, ensuring the future of the vivid research landscape in nuclear medicine in The Netherlands.

The new Dutch residency program provides a solid base for any new, young specialist to start a career in the exciting field of nuclear medicine, which, as for all medical specialties, also requires lifelong learning. Some of the nuclear radiology residents who have graduated are currently employed in major centers in The Netherlands and are running molecular imaging research programs on topics such as neurodegenerative diseases, orthopedics, or rheumatology. Many obtain job offers even before graduation, or they proceed with a fellowship to enhance their expertise in, for example, oncology or therapy.

Not everything has been easy in this transition period. Challenges for both nuclear medicine and radiology alike are posed by the collaboration between different professionals and by the variety of practices, ranging from academic to peripheral hospitals, on top of the teething problems of a new curriculum.

Houdini’s disappearance was an act of illusion, regarding which one should realize that nothing is what it seems at first sight. Since the start of the new program, the number of nuclear residents has increased continuously, and the first young nuclear radiologists perform adequately in a clinical (nuclear medicine) setting. For the future, it is important to note that the program is subject to periodic monitoring and updating.

It is up to the new generation of integrated imaging specialists to shape their own bright future and to prove themselves. At the same time, the older generation should give them a fair chance. Only then can we grow and profit from each other’s expertise and together face the challenges of high-throughput medicine and artificial intelligence, which may substantially alter the medical landscape and the skills needed to navigate it.
